# Immunogenicity to Biotherapeutics – The Role of Anti-drug Immune Complexes

**DOI:** 10.3389/fimmu.2016.00021

**Published:** 2016-02-02

**Authors:** Murli Krishna, Steven G. Nadler

**Affiliations:** ^1^Bristol-Myers Squibb Company, Princeton, NJ, USA

**Keywords:** immunogenicity, biotherapeutics, hypersensitivity, neutralizing antibodies, immune complexes, risk assessment, immunogenicity prediction, immunogenicity assay

## Abstract

Biological molecules are increasingly becoming a part of the therapeutics portfolio that has been either recently approved for marketing or those that are in the pipeline of several biotech and pharmaceutical companies. This is largely based on their ability to be highly specific relative to small molecules. However, by virtue of being a large protein, and having a complex structure with structural variability arising from production using recombinant gene technology in cell lines, such therapeutics run the risk of being recognized as foreign by a host immune system. In the context of immune-mediated adverse effects that have been documented to biological drugs thus far, including infusion reactions, and the evolving therapeutic platforms in the pipeline that engineer different functional modules in a biotherapeutic, it is critical to understand the interplay of the adaptive and innate immune responses, the pathophysiology of immunogenicity to biological drugs in instances where there have been immune-mediated adverse clinical sequelae and address technical approaches for their laboratory evaluation. The current paradigm in immunogenicity evaluation has a tiered approach to the detection and characterization of anti-drug antibodies (ADAs) elicited *in vivo* to a biotherapeutic; alongside with the structural, biophysical, and molecular information of the therapeutic, these analytical assessments form the core of the immunogenicity risk assessment. However, many of the immune-mediated adverse effects attributed to ADAs require the formation of a drug/ADA immune complex (IC) intermediate that can have a variety of downstream effects. This review will focus on the activation of potential immunopathological pathways arising as a consequence of circulating as well as cell surface bound drug bearing ICs, risk factors that are intrinsic either to the therapeutic molecule or to the host that might predispose to IC-mediated effects, and review the recent literature on prevalence and intensity of established examples of type II and III hypersensitivity reactions that follow the administration of a biotherapeutic. Additionally, we propose methods for the study of immune parameters specific to the biology of ICs that could be of use in conjunction with the detection of ADAs in circulation.

## Introduction

Immunogenicity is antigenicity in an inflammatory milieu resulting in a successful humoral response. With evolving protein therapeutic platforms, newer multi-domain therapeutics and the escalating investment of resources and technology toward the development of efficacious biologic drugs with minimal adverse effects, the phenomenon of immunogenicity has gained added relevance (1–7). Anti-drug antibodies (ADAs) can be elicited *in vivo* to a therapeutic and their detection has generally been equated as a measure of immunogenicity. The detection, reporting, and characterization of the ADA are done in a tiered manner after careful consideration of immunogenic risk factors. (8, 9). Most adverse effects consequential to ADA formation, such as pharmacological abrogation, impact on therapeutic exposure, or hypersensitivity reactions, are a consequence of formation of immune complexes (ICs) between the ADA and therapeutic protein. Their levels, kinetics of interaction, size, polyclonal diversity, distribution, and Fc-mediated physiological effects can be potentially translated to clinically observable adverse effects. This leads to the paradigm of immunogenicity where therapeutic exposure leads to ADA generation that in turn forms ICs that mediate adverse effects related to immunogenicity.

While the detection of such therapeutic specific IC from *in vivo* samples has remained analytically challenging, there are other biomarkers that mediate the interplay of the innate and adaptive immune responses and are potentially amenable to analysis. Such markers can reflect either the formation or the downstream effects of ICs. Molecular pathways underlying the immune response have been extensively studied to understand the pathophysiology of several autoimmune conditions (10) and it is most likely to be a question of degree and intensity of their involvement in an immune response to a therapeutic agent. The hope is to identify and describe some of these pathways whose analysis can be integrated pragmatically into the immunogenicity risk management process and consistently applied across the biotechnology industry for a shared learning across diverse therapeutic platforms. Considerable effort and progress has been made in identifying and mitigating risk factors from a therapeutic entity perspective – such as molecular engineering, formulation, biophysical character, route of delivery, and sequences with a propensity for binding to various MHC alleles. However from an *in vivo* perspective, there are host-specific phenotypic markers, some of which are polymorphic, with the distribution of Class II alleles in different populations being perhaps the most dominant example of such a host-specific characteristic (Table 1). These attributes in a host might explain the variability in ADA levels and their downstream effects or influence the formation and behavior of ICs. This would thereby represent the other half of the immunogenicity equation that needs to be considered as part of the total risk assessment package.

**Table 1:** **Factors to be considered during immunogenicity risk assessment of a biotherapeutic**.

Characteristics intrinsic to	Risk potential	Considerations
**Biotherapeutic structure and function**		
Structural and amino acid differences from native	High	Non-human sequences, low degree of humanization, and divergence of CDR sequences from germ line can break tolerance
	Low	Fully humanized sequences
Foreign protein to host	High	Enzyme replacement therapy to subjects with loss of function mutations in the proteins
Location of therapeutic target	High	Cell surface targets on immune cells that promote internalization, processing, and antigen presentation
Functional relationship with endogenous counterpart	High	No redundancy in endogenous protein function; high neutralization potential
	High	Endogenous protein is in low abundance, e.g., recombinant protein therapy, likely to be neutralized at high doses
	Low	Endogenous protein is abundant; e.g., monoclonal antibody based therapeutics
Mode of action	High	Immunomodulatory; check point inhibitors
	Low	Immunosuppressive treatments
Predictive analytical data	Varies	*In silico* analysis of amino acid sequences with high binding to class II MHC allelic variants
	Varies	*In vitro* T cell stimulation assays using PBMCs from healthy donors
**Biotherapeutic process and manufacturing**		
Chemical modifications	Varies	Oxidation, deamidation, isomerization have varying effects
Aggregation, denaturation	High	Repeat motifs cross-link B cell Ig receptors; unique conformational epitopes present in incorrectly folded denatured protein
Protein degradation	High	Structural variants can have unique linear and non-linear epitopes perceived as foreign
Contaminants and impurities	High	Host cell proteins, production, and purification process contaminants act as adjuvants
Post translational modifications	Moderate	α1-3 Gal, N glycolyl neuraminic acid, non-fucosylation are immunogenic risk factors
Formulation	Varies	Leachables in container, incompatibility with physiological pH, leading to product aggregation
**Clinical use**		
Route of administration	Varies	Risk highest: Inhalation > subcutaneous > intraperitoneal > intramuscular > intravenous
Dose	Varies	Higher doses more likely to increase risk
Frequency of administration and duration of treatment	Varies	Repeat dosage and prolonged exposure may either break or lead to tolerance
**Patient**		
Age and genetic predisposition	Varies	Pediatric vs. adult immune system, HLA allelic subtypes, genetic defects, polygenic traits underlying immune system competence
FcγR polymorphism, FcγRIIIa expression on CD4^+^ T cells	Varies	Expression levels, ratio, and cell type distribution of activating and inhibiting receptors
Preexisting antibodies and CD4^+^ T cells reactive to therapeutic	High	Cross-reacting auto antibodies, preexisting anti-PEG antibodies
Disease status and chronicity	Varies	Autoimmune or proinflammatory predisposition has a higher risk; chronic ailments necessitate prolonged exposure
Prior exposure to related or cross- reacting therapeutics	High	Common scaffold sequences or 2 unrelated therapeutics sharing the same altered Fc sequences or same linker
Concomitant medication	Varies	Co meds with immunosuppressive, such as methotrexate and steroids can reduce risk
Life threatening disease	Low	Higher tolerance to immunogenicity risk especially if no alternative therapy available
**Other associated data**		
Other therapeutic programs with similar indication or similar therapeutic platforms	Varies	Clinical data from other programs should be used as a guide for risk assessment
Non-clinical data	Varies	Non-clinical immunogenicity data are NOT predictive but useful for risk assessment if there is altered pharmacokinetic and efficacy data and immune adverse effects

Therapeutic molecules bound to cell surface protein targets may also attract circulating ADA leading to formation of ICs on cell membranes in tissues; adverse effects due to these are classified as type II reactions. ADA bound to drug in circulation gives rise to circulating ICs (CICs) and they can result in type III reactions. Regardless of their presence, ICs are relevant from two main points of analysis – their size and their propensity to activate complement. Both factors can drive formation of IC deposits and activation of inflammatory pathways. The size of the complex also affects Fc-mediated functions through interaction with a family of widely distributed activating Fc receptors (FcRs). FcR expression levels vary in individuals. In addition, there are naturally occurring polymorphisms in their type and distribution that influences Fc-mediated effects by the ICs.

This review will also summarize salient examples where ADA generation was related to clinically observed immune-related adverse effects and where characteristics of ICs could explain the molecular mechanisms underlying the clinical data. The case reports will focus on those where the clinical presentation represents an underlying type I or type II hypersensitivity.

## Biological Effects of Immune Complexes

### Activation of Fc Receptors

Fcγ receptors (FcγR) are widely expressed by several cell types in the hematopoietic system and play a primary role in pathological effects of ICs. High-affinity FcγR1 binds monomeric IgG but low affinity FcγRII (CD32) and FcγRIII (CD16) preferentially bind complexed IgG. They are present in multiple isoforms and can be classified as activating (FcγRIIA, FcγRIIIA) or inhibiting (FcγRIIB, FcγRIIIB) depending on their cytoplasmic motifs (11, 12). ICs typically with polyclonal reactivity and size exceeding a threshold will engage low-affinity FcγR. While larger aggregates and insoluble ICs are cleared by the mononuclear phagocytic system in the liver and spleen, moderate to smaller ICs persist in the systemic circulation for a longer period and engage low affinity FcγRIIa and FcγRIIIa triggering proinflammatory responses. Smaller soluble ICs formed near the zone of molar equivalence (Heidelburger curve) cause Type III hypersensitivity reactions that are manifested through three key mechanisms: (1) Deposition of ICs at anatomical sites with diffuse capillary network and filtering membranes leading to vascular thrombosis and localized inflammatory response, (2) Propensity of some ICs to opsonize and activate complement (discussed below), and (3) Ability of ICs to cross-link FcγR and complement receptors (CRs) on a variety of immune cells. Neutrophils account for 50–70% of circulating leukocytes and are often the first cells to be recruited at the site of IC deposition. They express two low-affinity FcγRs, FcγRIIA and FcγRIIIB, that can bind with both CICs and deposited ICs causing release inflammatory mediators (13). Besides FcγR, these cells also have CRs allowing them to bind to opsonized ICs. Perivascular and extravascular ICs first bind to FcγR on resident mononuclear cells (mast cells and macrophages), resulting in costimulation and a hyperactive state of these cells (11). FcR clustering from IC ligation triggers release of inflammatory mediators, and chemotactic chemokines that cause endothelial activation and further recruitment of neutrophils ultimately leading to IC-mediated tissue injury. These events serve to recruit and activate lymphocytes, monocytes, and dendritic cells, cells that play a vital role in the host adaptive immune response, thus, providing a mechanistic explanation of the innate immune system’s ability in influencing the immune system’s antigen-specific response.

**Figure 1:**
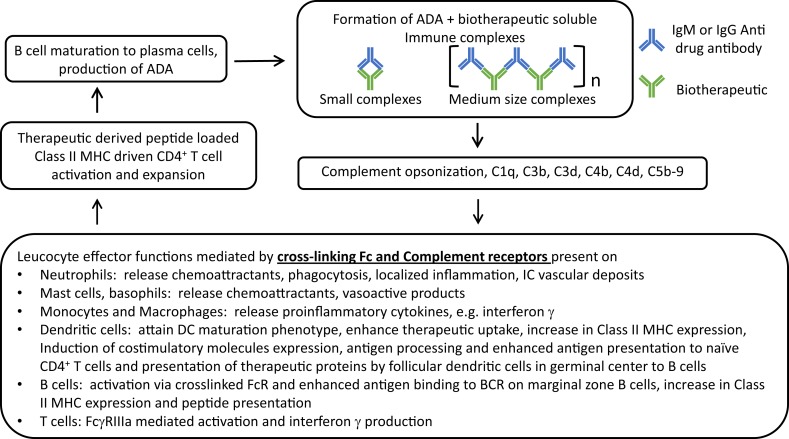
**The central role of immune complexes formed by biotherapeutic and ADA in the interplay between innate and adaptive arms of the immune system and exacerbation of ADA response**. ADA specific to CDR of a monoclonal antibody therapeutic is used as an example for ease of representing complex formation of varying sizes where *n* represents the number of cross-linked ADA Fc in IC; in reality ADAs are polyclonal with varying specificities.

Immune complexes can lead to further ADA production and potential IC formation. The cross-linked Fc portions of IgG molecules on an IC can: (a) bring together the FcR closer setting the stage for FcR activation, especially via ITAM bearing FcγRIIa and FcγRIIIa receptors. Monocytes, macrophages, and polymorphonuclear cells that express activating FcR produce proinflammatory cytokines in particular interferon (IFN)-γ that induces the expression of Class II MHC that further enhances the antigen uptake, processing, and presentation – eventually exacerbating a weak ADA response. (b) ICs carrying multiple therapeutic proteins can cross-link cognate B cell receptor (BCR) on the B cells producing the ADA, causing co-clustering of the BCR Ig alpha and beta chains leading to further activation of the B cells and more ADA (Figure 1).

### Activation of Complement Cascade

In addition to FcγR, complement also plays a critical role in mediating inflammatory processes downstream to formation of IC. ICs activate the complement cascade through the classical pathway by binding to complement component C1q via the Fc portion on ICs and unleashing a series of enzymatic activations and proteolytic events. The Fc of IgM ADA bearing ICs is very efficient in this reaction compared to IgG Fc. IgG_3_ ICs have greater propensity to fix complement than other IgG subclasses. Close proximity of IgG Fc on a complex mimics what IgM typically do best which is to efficiently activate complement system. Bringing the C1 complex close to one another lets them loose their inhibitor proteins and triggers the classical pathway to generate C3 convertase. Complement opsonized IC in circulation is a danger signal. A key primary event is the generation of complement C3 that is cleaved by C3 convertase giving rise to C3a and C3b. C3b binding to C3 convertase leads to generation of C5 convertase that cleaves C5 to C5a and C5b. Ultimately this leads to generation of an inflammatory response through the formation of anaphylotoxins, such as C4a, C3a, and C5a (14). Engagement of C5a to its receptor CD88 or the formation of sub-lytic C5b-9 complexes on lymphocytes can trigger GPCR signaling responses that are typically seen with activation pathways. C5a receptor engagement on mast cells and macrophages plays a critical role in modulating the ratio of activating and inhibiting FcγR on those cells. CRs on neutrophils promote their recruitment to sites of IC deposits and subsequent inflammatory damage in the tissue. Neutrophils by virtue of their FcR and CR play a critical role in initiating and sustaining IC-mediated immune responses (13). Several other cell types express CRs – but of note are B cells and antigen-presenting cells (APCs) where CRs interacting with opsonized ICs lead to their activation, increased antigen processing and presentation, and enhanced production of antibodies; this might be of significance allowing preexisting drug/ADA ICs to lead to higher ADA titers (Figure 1). ICs and complement can also directly influence T cell activation (15).

### Immune Complexes with Natural Antibodies

Preexisting antibodies that are predominantly IgM with low affinity and broad specificity are secreted by CD5+ B1-b cells (16). Such antibodies have been described against hinge regions and known to form complexes. IgM bearing IC tend to be bigger in size and more likely to be trapped in lymphoid organs where they may promote T–B cell interaction. These antibodies are typically produced without external antigenic stimuli and have evolved to enhance the immunogenicity against pathogens. There are genetic differences in humans with respect to their amount and specificity and some of these show up as preexisting reactivity to therapeutic molecules in immunogenicity assays. Diversity and specificity of adaptive immune responses to a therapeutic may be influenced by these antibodies. Any immunogenic protein entering the systemic circulation can bind either to preexisting natural antibodies, cross-reacting antibodies or to high-affinity class-switched antibodies elicited in response to the antigenic stimulus to form ICs.

### Promoting B Cell Maturation

Initial priming of naïve B cells results from the recognition of BCR to an antigenic epitope on the therapeutic molecule that is either conformational or linear. Biotherapeutic bearing ICs can also be taken up by peripheral dendritic cells that then migrate to the spleen and present antigens to the B cells in the splenic marginal zone. ADAs generated through these processes bypass T cell help and are typically of IgM isotype or low-affinity IgG. They can form ICs with the therapeutic molecule and FcR- and CR-mediated pathways can promote their uptake and processing by antigen-processing cells, including splenic marginal zone B cells. Presentation of Class II MHC bound therapeutic-derived peptides by these cells can eventually activate CD4^+^ T helper cells if their T cell receptors recognize them as foreign epitopes. Some of the antigen-primed B cells migrate to germinal centers of lymph nodes and can make contact with follicular T helper cells (T_FH_). Although epitopes from the therapeutic molecule that are recognized by T and B cells are fundamentally different, activated T cells secrete B cell growth factors that mediate T cell help and triggers formation of plasma cells from B cells, class switching, somatic hypermutation, production of high affinity ADA, and subsequent formation of memory B cells (17). Epitope spreading can also occur whereby novel epitopes formed within ICs with ADAs generated during a primary immune response are recognized by naïve B cells leading to their activation (10). FcR- and CR-mediated uptake of these ICs also induce antigen processing, and presentation leading to T cell activation and T cell-dependent ADA production by the B cells directed against an epitope different from the primary response. Follicular helper T cells are a specialized helper T cell population with a characteristic cell surface immunophenotype and their formation can be accentuated by the presence of IC. By virtue of homing to lymphoid organs, ICs can promote increased localized antigen concentration and presentation to the lymphoid cells.

### Effect on Follicular Dendritic Cells

Biotherapeutic drug bound ADA ICs above a certain size can bind through FcRs to follicular dendritic cells (FDC) present in the germinal center of lymph nodes. Furthermore, if complement opsonized, these ICs will be efficiently captured by germinal center FDCs via CR-1 and CR-2. Both pathways clearly enhance the loading, processing, and presentation of any immunogenic epitopes from the therapeutic protein and increase the opportunity of FDC recruiting T_FH._ This in turn will drive T cell–B cell interactions, a process necessary for B cell maturation to plasma cells. Presence of biotherapeutic molecules in the complex that are bound to either FcR or CRs on FDC in the germinal center will contribute to B cell engagement with the biotherapeutic, and their selection of BCRs with respect to specificity and affinity (6, 10). Thus, complement opsonization is a critical event on IC since it results in enhanced targeting and cell surface localization of the therapeutic antigen to FDC, and subsequent increased and efficient antigen presentation to B cell. This process is essential for B cells to produce a sustained and high titer anti-drug antibody response.

### Triggering Cytokine Signaling Pathways

Engagement of FcR or CR on cells though IC cross-linking results in the production of chemokines and growth factors that have a cascade effect on trafficking and growth of T and B cells. These soluble factors play a vital role, influencing the cross-talk between the innate and adaptive arms of immunity (18). Release of inflammatory cytokines that are routinely measured in various autoimmune disorders can also be indicative of drug-induced IC-mediated effects.

### Direct Influence of IC on T Cells

A subset of activated T_FH_ express FcγRIIIa that can deliver a Class II MHC-independent activation signal and induce them to produce IFN-γ. Recognition of ICs by FcγRIIIa results in T cell costimulation and may represent an alternate pathway linking the innate and adaptive arms of immunity (19).

## Clinical Outcomes Related to Immune Complexes

Adverse effects to biological therapeutics arise either due to an exaggerated pharmacological activity toward its intended target or due to immune-mediated toxicity resulting from a necessarily long-term therapy (20). Immune response to the drug resulting in ADA generation and its associated downstream effects covers part of the overall spectrum of immune-mediated toxicity. The pathological mechanisms underlying immunotoxicity are diverse and have been reviewed by others (21). The examples covered in this article will focus on two scenarios on how IC formed by the ADA with the drug translates to a poor clinical outcome; one where binding by neutralizing ADA results in abrogation of pharmacological activity of the drug and second where the ADA causes sequestration of the therapeutic by formation of an IC with the drug, and causing enhanced clearance resulting from recognition of ICs by FcR. Together, these mechanisms result in loss of efficacy, altered pharmacokinetic profile, cross-reactivity to endogenous versions of the protein, and hypersensitivity reaction manifested as infusion reactions to anaphylactic reactions (22–25). In general, these examples cover ADA responses to enzyme replacement therapeutics where the therapeutic is seen as a foreign protein in a host expressing the mutant allele, or to some recombinant protein therapeutics that result in breakdown of tolerance to the endogenous counterparts, and to some of the anti-TNF-α agents and few other monoclonal antibody therapeutics, such as natalizumab (anti alpha4 integrin), and cetuximab (anti EGFR). It is important to emphasize on the perspective that while most biologics do elicit some titer of ADA with varying duration, a majority of them do not have overt clinical side effects. This may relate to the fact that formation of an IC may not necessarily relate to poor clinical response if the ADA levels are transient or low affinity and if there are enough active therapeutic molecules at the site of action to achieve a pharmacological response (26). It is possible that disease states can have an influence on the formation of ADA and, therefore, formation of IC. An autoimmune predisposition may result in an exaggerated immune response although no rigorous studies have been conducted comparing immunogenicity data in patients with rheumatoid arthritis or inflammatory bowel disease to patients with no underlying proinflammatory state. However, results with a drug such as belimumab, an antibody used for the treatment of lupus, a prototypical antibody-mediated disease, do not reveal higher rates of immunogenicity vs. antibody therapeutics in other indications. Other considerations that could impact clinical immunogenicity outcomes include mechanisms of action of immunomodulatory therapeutics that include binding and uptake of the therapeutic molecule by APCs, and subsequent activation and maturation of APCs, events that would clearly predispose to immunogenicity.

There is a considerable body of literature on immunogenicity to anti-TNF-α biologics and their role in immunopharmacological adverse effects; this has been reviewed elsewhere (1, 24, 25, 27–32). ADA responses to adalimumab and infliximab have been linked to clinically manifest adverse effects; by contrast, ADA responses to other anti-TNF-α therapeutics, such as etanercept, golimumab, and certolizumab, have not conclusively showed a link to reduced clinical response (1). A significant percent of patients with rheumatoid arthritis receiving adalimumab and infliximab go on to develop ADA within the first 6 months of therapy (25, 28, 29). Likewise development of ADA and its role in serious side effects have been described for recombinant biologics, such as IFNs (33–35) erythropoietin (36), thrombopoietin (37), and Factor IX (38). Many of the adverse effects are most likely mediated by formation of ICs with the drug.

## Factors Modulating Impact of Immune Complexes

Immune complexes themselves are heterogeneous and vary with ADA titer, isotype distribution, specificity, affinity to the drug, size of the circulating complexes, and antigen. These attributes influence how their formation translates to clinical consequences.

### Specificity

In terms of specificity, an ADA may either recognize the functional portion of a drug leading to abrogation of its pharmacological activity and thereby become a neutralizing antibody (NAb) or bind to pharmacologically irrelevant portions of the drug. While NAbs by virtue of their specificity prevent therapeutic target recognition, both NAbs and other binding antibodies can influence therapeutic exposure by either hastening the clearance of the drug (clearing antibodies) or enhance its circulating half-life (sustaining antibodies) (39). These two classes of ADAs may vary in titer and duration and thereby influence the outcomes. Patients treated with IFN-β had earlier onset, higher titers, and longer persisting non-neutralizing ADA than neutralizing ADA (35, 40). ADA responses to anti-TNF-α agents have varied with the specific agent; ADAs elicited in adalimumab- and infliximab-treated patients were predominantly NAbs (41), while those receiving etanercept have mostly been non-neutralizing ADAs (1). Recombinant erythropoietin and thrombopoietin therapies have resulted in ADAs that both cross react and functionally neutralize the endogenous versions often leading to serious clinical adverse effects (36, 37). Changes to the biophysical characteristics of the therapeutic protein often leading to aggregation can potentially break tolerance and lead to ADA formation reacting to the endogenous proteins. Likewise recombinant IFN-β1a and IFN-β1b therapy-induced ADA that was cross-reactive with endogenous IFN-β (42). Functional characterization of patient-derived NAbs to adalimumab (30, 41) by single B cell cloning revealed some interesting observations; there was clonal diversity within the anti-idiotypic population of NAbs and most bound to the drug with high affinity. The proclivity to form NAbs might be related to two factors: (a) the degree of divergence from germ line CDR sequences induced by extensive somatic hypermutation during therapeutic antibody engineering and (b) the number of idiotopes located within the drug’s target recognition domain. Despite minimal divergence from germ line sequence, adalimumab elicited higher than expected titer of NAbs; this was postulated due to extensive regions in its CDRs that conferred TNF-α recognition, thus, making it more likely for ADAs with such specificity to interfere with target recognition.

Multidomain therapeutics pose new challenges in dissecting the specificity of a polyclonal ADA response. Targeted cytokines made of an active cytokine covalently linked to Fab and Fc and antibody drug conjugates made with active payloads linked to an IgG through linkers are being developed as treatment modalities for cancer. These complex molecules can potentially elicit ADA reactivity to a variety of epitopes on the therapeutic and epitope characterization of ADA requires careful reagent development (43).

### Size

Restriction of immunogenic specificity to a small region has an important bearing on the behavior of IC. The tighter the restriction, greater is the likelihood of forming dimeric complexes of drug and ADA or formation of small ICs since steric hindrance of one ADA bound to the drug will restrict further ADAs from binding. This was indeed the case with adalimumab-specific IC formed in three representative patients’ samples that were studied by sucrose gradient fractionation and shown to be small and mostly dimeric in nature (41). However a more recent study by the same group also showed the presence of larger multimeric ICs formed by ADAs derived from B cells isolated from patients who were ADA positive to adalimumab (30). The implications of IC size may have a bearing towards altered pharmacokinetics; smaller complexes can persist for longer durations since they do not meet the minimal threshold to engage low-affinity FcR and will probably recycle the drug back into circulation through the FcRn pathway and larger complexes can be cleared faster and efficiently by low-affinity FcR recognition that mediate uptake and breakdown of the complexes. Infliximab bound ADA ICs were studied in cynomolgus monkeys (44) and human subjects (45), and showed formation of small complexes probably dimeric in nature and larger complexes that were tetrameric and higher. Larger complexes clear from the circulation faster than the smaller complexes (44), while smaller complexes could persist in circulation for up to 2 weeks (41). A comparative analyses of ICs resulting from different anti-TNF-α treatments has shown formation of smaller complexes with etanercept and larger complexes with adalimumab and infliximab (46). While larger complexes are picked up earlier by macrophages and cleared faster, they are also less likely to be available to trigger inflammatory pathways. Instead complexes that are opsonized by complement are more likely to be pathologic since they tend to be soluble, stay longer in systemic circulation, and would also engage B cells through both CR-2 and BCR. Such ICs can engage B cells through FcR, BCR, and CR-2 receptors, and deliver a potent signal.

### Initiation and Potentiation of Immune Response

Specific recognition of a drug by a B cell is an early and necessary event in immunogenicity and T cell recognition of therapeutic-derived peptides is necessary for B cell activation and maturation to plasma cells capable of producing high titer antibodies. ADAs are made by B cells utilizing T cell-independent and -dependent pathways. In general, drugs that tend to aggregate or multimerize can cause presentation of repeating epitopes that can cross-link BCR and clustering leading to their activation without T cell help (47–49); aggregation may also distort conformation resulting in novel B cell epitopes (50). Composition of the formulation or contamination with leachables and extractables may lead to aggregation and be a risk factor for immunogenicity (51, 52). ICs with drug molecules trapped within them may also be engulfed by dendritic cells and be presented to splenic marginal zone B cells triggering another pathway of T-independent pathway of ADA (53). ADAs generated by this pathway are typically low affinity and IgM. However, mechanisms that allow the drug to get internalized, processed, and presented by Class II MHC on APCs and the presence of T cell epitopes in the primary sequence of the drug will promote T-dependent activation of B cells (54–56). Marginal zone splenic B cells or dendritic cells localized within tissue-specific compartments important for surveillance may capture and process aggregated therapeutic or ADA bound complexes of therapeutic leading to antigen presentation to T cells. These pathways illustrate how a seemingly low-affinity T-independent generated ADA from a primary immune response could lead to formation of a drug–ADA IC which in turn through uptake by Class II bearing B cells or APCs could activate T cells and thereby leading to further B cell activation, epitope spreading, clonal expansion, affinity maturation, and generation of higher affinity ADA (Figure 1) (10, 16). The ICs formed early may also involve preexisting circulating IgM or IgG antibodies that recognize the biotherapeutic. Examples of these include antibodies recognizing polyethylene glycol (PEG) a frequently used covalent adduct for some biologics (57, 58) and also N-glycolylneuraminic acid a non-human sialic acid that gets incorporated into therapeutic proteins made from non-human mammalian cells (59). While aggregated therapeutics can be internalized through macropinocytosis or phagocytosis or receptor-mediated endocytosis into APCs such as immature dendritic cells present locally at the site of administration, IC bearing drug molecules can also be taken up through Fc gamma receptors; further potentiating drug processing and presentation by class II MHC on those APCs (60). This might also explain the observation that subcutaneous route of administration causes more immunogenicity than other routes probably due to the localization and proximity of the therapeutic to APCs such as Langerhans cells present locally. The importance of IC in exaggerating an immune response is further underscored if the complexes get opsonized with complement. Complement can potentiate uptake of the ICs through CR-1 and CR-2 receptors (61) and also play a role in the maturation of immature dendritic cells and expression of costimulatory molecules that eventually are required for T cell activation (42, 62, 63).

In some instances, immunogenicity is driven by breakdown of self-tolerance than a response to foreign epitopes. Several factors lead to this breakdown, including repeated exposure to a biotherapeutic necessitated by the chronicity of the illness. At a molecular level, the coupling of T cell epitopes with self-antigens and a potent mechanism to ferry this therapeutic into dendritic cells with a mature phenotype combine together to break tolerance (49, 52).

### Isotype and Subclass

T cell help results in class switching and higher affinity ADAs typically of the IgG isotype (64). Anti-TNF-α agents such as infliximab and adalimumab that are known to elicit higher titers of NAbs tend to be more IgG_1_ and IgG_4_. The skewing of an ADA response to include more IgG_4_ subclass production can be seen over a period of time covering persistent ADA titers and may be due to repeated exposures to an immunogenic therapeutic (65–67). IgG_4_ ADAs have also shown to have higher neutralizing capability than IgG_1_ ADA probably due to the prolonged nature of antigen stimulation and repeated rounds required for affinity maturation (64, 68). However, this may be dependent on the nature of the therapeutic agent and there are instances with recombinant interferon treatment where most of the neutralizing ADAs were IgG_1_ and not IgG_4_ (42). By contrast, IgM ADAs are considered to be of lower affinity and transient; any IgM bound therapeutic ICs can have more potent consequences than IgG complexes due to its multivalency and propensity to cross-link FcRs and fix complement. IgM type ADAs have been demonstrated with interferon-α 2b therapy (69) and TNF-α therapy; infusion reactions in some patients receiving infliximab were associated with presence of both IgE and IgM ADA in the serum (70). IgG_1_ and IgG_4_ antibodies are typically seen to protein antigens, IgG_2_ subtypes are induced by glycosylated epitopes (65).

### Complement Activation

ADA-therapeutic IC activate complement. Only IgG and IgM isotypes are known to activate complement; the pentameric structure allows low levels of IgM bearing complexes to efficiently and readily activate complement. IgG_1_ and IgG_3_ subclasses are more potent complement activators than IgG_2_ and IgG_4_ (71). Complement opsonization by ICs can further potentiate a weak ADA to a higher titer ADA response. Products of complement activation directly or indirectly modulate dendritic cells and the humoral and cellular arms of the adaptive immune response. APCs bearing CR-1 and CR-2 can internalize complement opsonized ICs resulting in processing and presentation of therapeutic protein derived peptides to T cells (14, 63). In addition, complement proteins modulate T cells leading to their activation and differentiation, which influence B cell antibody production and memory cell formation (14). A study on the treatment of 19 patients with relapsing-remitting multiple sclerosis with recombinant interferon-β showed interaction of ADA with the therapeutic interferon-β forming ICs and subsequent complement activation (42). IgE independent hypersensitivity reactions are mediated by IC-mediated complement activation and the acute phase reactants released downstream to complement activation (72).

### Hypersensitivity Reactions

Fundamental to any type of hypersensitivity reaction is the formation of an IC of an ADA with its cognate partner. Such phenomena have been studied in both pre-clinical and clinical models (1, 4, 5, 24, 38, 73). In type I hypersensitivity, IgE isotype ADAs are formed during an initial response and upon repeat exposure to the therapeutic agent IgE bound complexes bind and cross-link to Fcϵ receptors on basophils and mast cells leading to acute degranulation, release of histamine, and manifestation of anaphylactic reaction. IgE-mediated anaphylaxis was documented in some patients receiving infliximab (70). Such reactions can also be potentially fatal. Atypical anaphylaxis can also be triggered with IgG isotype ADA when such IgG bearing ICs formed after a second exposure cross-link Fcγ receptors on neutrophils leading to activation and release of platelet activating factor supposedly more potent than histamine (74, 75). Type III hypersensitivity involves formation of large ADA/therapeutic protein complexes in the correct stoichiometry that do not get cleared but instead precipitate and deposit in tissues rich in filtering membranes made of fenestrated endothelium, such as kidneys, synovial membranes, and the choroid plexus. Animal necropsy studies have shown deposits are typically formed in post capillary venules. Further downstream tissue damage is mediated by complement fixation and activation or Fc-mediated inflammatory sequelae. This type of hypersensitivity is highly dependent on the ADA/drug ratio in the complex and might explain why it is relatively rare and variably seen despite the ease of forming CICs. The pathology is multifocal and accompanied by cell death and compromised organ function. Venous and arterial thromboembolic phenomena with high titer ADA have been described in three patients receiving adalimumab treatment. The underlying pathology in these patients is related to IC formation with adalimumab ADA and its downstream effects. Presence of anti-adalimumab ADA in a cohort of patients in the same study was associated with higher risk of developing thromboembolism (76). Clinical manifestations and humoral and cellular immunity pathways associated with drug-induced hypersensitivity reactions have been well reviewed elsewhere (73).

## Analytical Challenges in the Assessment of Immune Complexes

Most assays used for screening for the presence of ADA utilize a bridging assay format that allows two labeled drug molecules to be bridged by an ADA; in some instances, the ADA is captured first by drug attached to the surface of a plate and then detected by a second antibody. The bridging format depends on the bivalency or multivalency of the ADA and may fail to detect IgG_4_ subclass ADA that typically starts to appear much later after prolonged exposure to drug. Formation of interchain disulfide bonds in the hinge region of IgG_4_ molecules is inefficient and could result in formation of either IgG_4_ halfmers or heterodimers; such ADA molecules presenting as monovalent binding molecules would be unable to form a bridge between two drug molecules (66). Cross-binding of labeled drug molecules in presence of rheumatoid factor, anti-allotypic antibodies, or soluble target and epitope masking on labeled drug molecules as in a solid-phase capture reagent are some reasons for false-positive and false-negative data (77). Comparative analysis of ADA responses to different therapeutic agents to a common target are influenced not only by intrinsic differences between the therapeutic agents, and lack of consistency in study design, influence of immunosuppressive comedications, sampling time, and methodologies but also profoundly influenced by the semi-quantitative nature of the immunogenicity assay, differences in the assay formats used to measure the ADA and most importantly the assay’s tolerance to levels of circulating therapeutic (78–80). Several techniques, such as acid dissociation, are used to overcome the drug interference in ADA detection and many of these methods are laborious and might alter properties of ADA that affect their detection in assays (79, 80). This affects interpretation of ADA levels against any given therapeutic determined at different time points in the same study population or in different disease states or in subjects with different routes of administration of the therapeutic. More importantly, choice of reagents, controls, and assay design will limit any conclusions that can be made from studies attempting to compare immunogenic profiles of different therapeutic agents or modalities of treatment.

Attempts to measure both bound ADA within ICs and free ADA as a representation of total ADA levels have been limited. A versatile and elegant generic method of measuring bound as well as total ADA in cynomolgus serum samples dosed with human Fc-based therapeutics was described by Stubenrauch et al. (81). They were able to differentiate total from bound ADA by comparing samples spiked with drug *in vitro* to samples that were not. The method depends on the availability of well-characterized reagents with specific reactivity to human IgG or cyno IgG but not cross-reacting to both. The method demonstrated value in picking up ADA responses earlier in the study and also detecting ADA even at drug levels that inhibited their detection in the traditional bridging assay format. Such methods may not be feasible with clinical samples where cross-reactivity with normal human IgG would adversely affect the assay. Methods developed by our group took advantage of a non-Fc bearing therapeutic protein to detect drug–ADA ICs in cynomolgus samples and arrived at similar conclusions that ADA bound in ICs significantly limited their detection by bridging assay format (82). A key consideration for any assay designed to detect drug-specific ICs would be the choice between using covalently or non-covalently coupled drug and ADA complexes for method development and calibration. While covalent coupling in the ratio of 1 ADA to 1 drug molecule provides consistency across assays, it does not fully represent the diverse range of ICs seen in actual samples and, therefore, may not provide an accurate estimation (82). In another study, formation of infliximab and anti-infliximab IC in human subjects was tracked and characterized by infusing radiolabeled infliximab to four patients, two of whom were non-responders. Whole-body imaging and sucrose gradient separation of serum samples revealed formation of higher ADA levels and large ICs in non-responders with faster clearance of infliximab and uptake of radiolabeled drug in liver and spleen (45). As discussed earlier, larger ICs are generally cleared faster through FcR recognition and internalization by cells of the reticulophagocytic system.

Several groups have used some unique attribute of ICs in general to estimate total ICs; these include complement binding and there are commercial kits (Quidel MicroVue, CIC-RAJI EIA, Cat #A002) that make use of this property (83). CICs bind on red blood cells through CR-1, which is a mechanism for their removal. It is a marker that can be measured by flow cytometry and varying expression may be associated with clearance of the complex; lower expression may be associated with reduced clearance and increased risk of IC-mediated adverse effects. Other assays have been described measuring circulating and opsonized complement (84). Commercial kits that measure C3a and C5b-9 (Quidel MicroVue SC5b-9 Plus EIA) can be used as indicative of complement activation. These have been reviewed by others (5). Higher levels of C3a and C5b-9 are indicative of complement activation. These assays are not routinely performed and lack standardization in procedures across laboratories. International standards for complement assays have been published and need to be uniformly adopted (84). Measurement of complement products directly bound to the drug-specific IC is physiologically more relevant and eliminates interference due to variations in protein metabolism, or other autoantibodies. Such technologies are being evaluated here and elsewhere wherein specific immunocapture of total drug from biological samples followed by mass spectrometric analysis of signature peptides specific to complement components on the C1q and C5b-9 that might have covalently reacted with the IC would reveal the extent of opsonization. Using the same approach, one could even isotype the distribution of IgM and IgG subtypes in the IC. These methods are distinct from some of the currently available isotyping platforms that only study free ADA in the sample.

It should be emphasized that assays that identify CICs or products of complement activation do not provide specific information on therapeutic bound to ADA ICs and CICs in general can be elevated following infection, or patient’s disease state such as autoimmune condition. Identification of drug-specific ICs would need reagents with exquisitely defined specificities. Using samples from animal studies might be a better approach than human study samples to study ICs with therapeutic humanized antibodies. ICs may also be present in serum of normal healthy adults and not all drug–ADA complexes are necessarily pathological; those that are significant would be dependent on (a) their size and binding to low-affinity FcγR on phagocytic cells causing faster clearance of the therapeutic and (b) their ability to opsonize and activate complement leading to inflammatory sequelae. These outcomes are clinically relevant. Our work on ICs in cynomolgus monkeys showed that only a fraction of total drug-specific ICs were bound to the low-affinity FcγR and despite high levels of drug-specific ICs in circulation, there was no evidence of complement-mediated activation or inflammatory cytokine elevation (82) in the monkeys. With this in perspective, it would be more meaningful to assess size, clonality, IgG subtype, or IgM composition of the complex and any evidence of ongoing opsonization – all factors that could influence FcγR binding or complement activation. Demonstrating just the presence of a drug and ADA complex may not be any more helpful from a clinical standpoint than showing presence of ADA in the serum.

The use of immunohistochemistry techniques using specific reagents to follow drug-induced ICs along with colocalization of terminal complement present around cynomolgus monkey tissues from toxicology studies have also been described (5). Likewise morphological data to support drug-induced hypersensitivity reaction and IC deposition around sub-endothelial and sub-epithelial regions of the glomeruli can be enhanced through the use electron microscopy (5). It is critical that any technique studying the distribution of ICs or activated complement products in any biological material must demonstrate the presence of the therapeutic molecule with specific reagents to support pathology, resulting from drug-induced ICs. Such interpretations should not be confounded by reagent cross-reactivity to endogenous IgG. Reagents that bind to human IgG that is a commonly used therapeutic platform must be cross adsorbed to ensure no binding with monkey or the relevant species’ IgG. In order to broaden our understanding of phenomena linked to formation of ADAs, comparative data across different therapeutic platforms and disease backgrounds will be important and will, therefore, require some minimum consistency in controls and procedures.

## Toward a Road Map for *In Vivo* Risk Assessment for Immunogenicity

There are several factors that make individuals in a population respond distinctly to a therapeutic and also play a possible role in the immune pathways. Allelic polymorphism in MHC plays a key role in T cell activation and plasma B cell proliferation and differentiation leading to production of ADA (17). But these studies have been limited only to a few commonly expressed alleles and focus solely on the cellular immunity pathways. The ability of drug-specific ICs to fix complement, trigger ADCP or ADCC can influence long-term outcomes. These variables are related as discussed earlier to the clonality of ADA and thereby the size of the complex, the isotype – with IgM and IgG_3_ being most efficient in fixing complement and also the microenvironment of drug interaction with the target. Tissue microenvironment can comprise stromal inflammation, localized concentration of cytokines, acute phase reactants, and presence of proteases and granzymes secreted from T cells and neutrophils that can lead to protein degradation and epitope generation. FcR polymorphisms within individuals in a population specifically related to the mutations that affect IC binding would significantly affect IC clearance and function of various immune cells (12). In this regard, it would be interesting to develop and use flow cytometry techniques to specifically immunophenotype-activated T and B cells in samples that test positive for ADA and identify FcγRIIb variants and their distribution on B cells and FcγRIIIa on T cells. These markers need to be compared to their baseline expression and correlated with other early and late activation markers of immune cell activation. Members of the Siglec family of cell surface proteins, also called CD33, show variation between individuals and are known to regulate immune activation of T and B cells (85). Preexisting antibodies or CD4^+^ T cells reactive to specific epitopes on the drug molecule could play a role through formation of ICs with the drug that can boost the immune response or cause epitope spreading as discussed earlier. Preexisting antibodies to N-glycolylneuraminic acid (59) and PEG (58) have been implicated in higher immunogenicity to biotherapeutics. Any comparative assessments of immunogenicity profiles of different classes of therapeutics should be considered not only in the context of underlying disease state as mentioned earlier, but also whether patients were treated with immune modulating comedications. Anti-TNF-α drugs are used in rheumatoid arthritis and Crohn’s disease/Ulcerative colitis patients. Approximately, 80% of patients in all disease indications use anti-TNF-α drugs in combination with an immunosuppressive such as methotrexate. Hence, it is likely there will not be a difference in immunogenicity across these different diseases.

Clearly, for any *in vivo* evaluation to be of value for immunogenicity risk assessment, there must be a few criteria. First, the biological characteristic under question must be responsive to perturbation of the immune system, when compared to a predefined baseline profile and preferably must have a mechanistic connection with clinical adverse effects. As emphasized earlier, there has to be underlying variations in their levels of expression or in the expression of alternate allelic forms or variants between individuals in a population that might modulate the immune response to the therapeutic. Their sampling must be easy and made accessible for exploratory work, reagents must be readily available and well characterized and most importantly the assays should be well standardized across multiple studies, diseases, sites, and therapeutic platforms for meaningful comparative research. Lastly, the data have to be amenable for multivariate analysis to make any correlations with ADA titers, its kinetics, and population frequency and with clinical outcomes to be of any predictive value. Hopefully systems pharmacology approaches can provide modeling of subtle person-to-person, and disease-to-disease variations that make it more or less likely for an ADA response to form, and demonstrate how this information can be integrated within the context of ADA risk assessment. Such an endeavor might benefit from an open source medium of exchange of materials and data through a consortium. Significant attempts have been made drawing together collaborators across the biotechnology industry, academia, and regulatory agencies in the ABIRISK consortium to standardize and define end points, materials and methods, and share the learnings (86).

## Conclusion

Most recombinant engineered therapeutic proteins are administered to patients as repeated doses, over their lifetime. Generation of ADA is a potential outcome to almost all such therapeutics. These can form ICs with the therapeutic which in turn can drive more ADA formation. Clinical consequences of ADA make a compelling case for early IC formation that is an important consideration whether or not a long-lasting, pharmacologically meaningful ADA response will form. With the advent of personalized treatment, there will be a greater need to monitor underlying differences between individuals who are reactive to a therapeutic and how they impact either their response to treatment or their manifestation of any immunological adverse effects. Clinical decisions in routine practice rarely make use of information on the patient’s immune response to a therapeutic as a basis to understand poor therapeutic response or an unexpected adverse effect; to some extent, this has been due to limitations to identify the right dose of the drug required to neutralize the target in the presence of ADA, challenges in ascertaining total amount of ADA, and a general lack of immunogenicity assessments in patients to investigate failure of response after a drug has been approved for market. The semi-quantitative nature of routine ADA assays, and diversity in assay formats and platforms also make it difficult to make meaningful comparisons in immunogenicity data across multiple therapeutics that a clinician might consider for treatment of a specific disease. Instead, the approach has been to switch to an alternate treatment (77) or take advantage of other immunomodulatory agents that negate the titer and impact of ADA. Patients with autoimmune conditions, such as rheumatoid arthritis, spondyloarthritis, and Crohn’s disease, and treated with anti-TNF-α agents have benefited from concomitant methotrexate treatment (87). Clinicians should be aware that formation of ICs and the accompanying risks they entail could persist as long as the same treatment continues unabated even with symptomatic remediation of adverse effects. Despite understanding and mitigating risk from the therapeutic aspect, such as selecting the right molecular structure and sequence, formulation, preventing aggregation, and choosing appropriate routes of dosing, the question on why some individuals and other do not develop clinically significant ADA titers persists and has also been discussed (3). Some aspects, such as genetic differences, HLA allelic variants, underlying disease state, and presence of preexisting reactive T cells and natural antibodies, might shed additional clarity on this variability. However, the formation and contribution of ICs is central to most of the downstream sequelae that are seen following development of ADA. Of particular importance, is the role of IC with low affinity non-neutralizing binding ADA; these IC through FcR interaction, complement fixation and subsequent downstream effects on APCs and B cells, further potentiate the ADA response leading to epitope spreading of ADA, formation of neutralizing antibody, ADA affinity maturation and further IC formation. The study of IC biology specific to the therapeutic will shed more light on the role and relationship of ADA to clinical outcome measures.

## Author Contributions

All authors listed, have made substantial, direct and intellectual contribution to the work, and approved it for publication.

## Conflict of Interest Statement

The authors declare that the research was conducted in the absence of any commercial or financial relationships that could be construed as a potential conflict of interest.
